# Changes in Microbial Community Diversity and the Formation Mechanism of Flavor Metabolites in Industrial-Scale Spontaneous Fermentation of Cabernet Sauvignon Wines

**DOI:** 10.3390/foods14020235

**Published:** 2025-01-14

**Authors:** Chunyan Bai, Boyuan Fan, Jinmei Hao, Yuan Yao, Shiming Ran, Hua Wang, Hua Li, Ruteng Wei

**Affiliations:** 1 College of Food Science and Engineering, Shanxi Agricultural University, No. 1, Mingxian South Road, Taigu District, Jinzhong 030801, China; 20210707628@stu.sxau.edu.cn (C.B.); 20233650@stu.sxau.edu.cn (B.F.); 20233688@stu.sxau.edu.cn (J.H.); 20220707807@stu.sxau.edu.cn (Y.Y.); 20210707640@stu.sxau.edu.cn (S.R.); 2 Beijing Hongxing Liuquxiang Co., Ltd., Liuquxiang Branch Company, Industrial Zone, Qixian, Jinzhong 030900, China; 3 Xinjiang Deyun Xingtai Agriculture Co., Ltd., No. 32, Dingxin Road, Fuhai, Altay 836400, China; 4 College of Enology, Northwest A&F University, No. 22, Xinong Road, Yangling, Xianyang 712100, China; wanghua@nwafu.edu.cn (H.W.); lihuawine@nwafu.edu.cn (H.L.)

**Keywords:** high-throughput sequencing, indigenous microorganism, spontaneous fermentation, community function, metabolic pathway

## Abstract

The key flavor compound formation pathways resulting from indigenous microorganisms during the spontaneous fermentation of wine have not been thoroughly described. In this study, high-throughput metagenomic sequencing and untargeted metabolomics were utilized to investigate the evolution of microbial and metabolite profiles during spontaneous fermentation in industrial-scale wine production and to elucidate the formation mechanisms of key flavor compounds. Metabolome analysis showed that the total amount of esters, fatty acids, organic acids, aldehydes, terpenes, flavonoids, and non-flavonoids increased gradually during fermentation. Enrichment analysis indicated that metabolic pathways related to the synthesis, decomposition, transformation, and utilization of sugars, amino acids, and fatty acids were involved in the formation of key flavor compounds in wine. Metagenomic analysis revealed that *Saccharomyces*, *Hanseniaspora*, *Zygosaccharomyces*, *Wickerhamiella*, *Lactobacillus*, and *Fructobacillus* were the dominant taxa during spontaneous fermentation. They were significantly positively correlated with organic acids, fatty acids, esters, phenols, aldehydes, terpenes, and phenols. In conclusion, this research provides new insights into the metabolic pathways of key flavor compounds formed by indigenous microorganisms during wine fermentation.

## 1. Introduction

As a commercial product with social and cultural significance, wine, particularly wine with terroir characteristics, is favored by consumers. It is generally agreed upon that the idea of *terroir* is derived from the interaction of the soil, climate, environment, topography, and other factors that have a significant impact on the quality of the grapes in a particular region that has its own viticulture and winemaking practices, which affect the wine sensory style and quality within the region [1]. In this sense, apart from these factors, a substantial amount of research has highlighted that native microbiota can contribute to the wine-making process in specific regions [2,3]. Wine fermentation involves a sequence of complex biochemical reactions of yeast and bacteria. By producing metabolites, the majority of weak- and strong-fermenting yeast species alter the chemical environment and fermentation process of wine, ultimately affecting the wine’s color, aroma, and taste [4]. The amount and length of these species’ presence throughout the alcoholic fermentation process determines their contribution to the characteristics of the wine [5].

In most cases, *S. cerevisiae* prevails during the wine fermentation process. Nonetheless, some non-*Saccharomyces* yeasts and bacteria actively participate in the development of unique wine characteristics. Non-*Saccharomyces* yeasts dominate during the initial phase of spontaneous fermentation, including species like *Kluyveromyces*, *Pichia*, *Hanseniaspora*, *Candida*, and *Torulaspora*, which are detectable at various phases of fermentation, contingent on the region, grape species, and actual vinifying process [4,6,7]. With the continuation of fermentation and the increase in the ethanol concentration, most non-*Saccharomyces* yeasts cannot survive, while *S. cerevisiae* becomes the dominant species and completes alcoholic fermentation, which is a common feature of all wine-making processes [8]. In addition, the bacteria *Lactobacillus*, *Leuconostoc*, *Oenococcus*, and *Pediococcus* are also implicated in malolactic fermentation and wine maturation. The former refers to a biological conversion process of malic acid into lactic acid, which elevates the wine pH, in addition to affecting the microbiome and flavor stability [9]. In the meantime, grape infection and colonization by fungal genera, such as *Penicillium*, *Aspergillus*, and *Botrytis*, have been suggested. These fungi produce metabolites during fermentation that can alter the environment in which yeasts and lactic acid bacteria grow [10,11]. For example, noble wines, which are made from *Botrytis cinerea*-infected noble-rot grapes, display a rich honey, floral, and fruity aroma, and high polyphenol and sugar content [12,13]. To control the fermentation parameters effectively and minimize the risk of spoilage and unforeseeable alterations in wine flavor, contemporary winemaking often employs commercially available active dry yeast. Nonetheless, certain evidence suggests that the continued application of commercial yeast drastically decreases indigenous yeast variability and reduces the wine’s aromatic sophistication [14]. Although the influence of indigenous non-*Saccharomyces* yeast strains on wine aroma has been extensively reported, the role of indigenous microorganisms in regard to the mechanism for wine flavor formation during the spontaneous fermentation process has never been systematically explored.

The present work attempts to elucidate the succession of flavor metabolites during the wine fermentation process, as well as the functionality and structure of related microbial communities, identifying the dominant populations implicated in fermentation and their interplay with core flavor metabolites, by integrating metagenomics and metabolomics assessments. In addition, gene prediction and annotation during the fermentation process were performed to clarify the possible functional role of the dominant population that are perhaps implicated in flavor formation.

## 2. Materials and Methods

### 2.1. Samples

The present study was conducted in 2023 at a winery in Qingtongxia, Ningxia, China, where the raw material used was Cabernet Sauvignon grapes at optimal ripeness (255 g/L total sugar, 4.6 g/L titratable acidity). Prior to being filled with grapes, we cleaned and disinfected the fermentation tank and space environment. The grapes were crushed and loaded into the tank, filling 75–80% of the 5 m^3^ tank capacity. Instead of using the commercial yeast inoculum, spontaneous fermentation was conducted at temperatures of 24–26 °C. Triplicate fermentation broth samples were gathered (following broth homogenization in the tank; samples were acquired from the lower, middle, and upper tank areas) at five time points: day 1 (A, destemmed, crushed grapes prior to fermentation), day 3 (B, Baumé meter reading dropped to 0.003–0.005), day 5 (C, Baumé meter reading dropped to 0.03–0.04), day 9 (D, Baumé meter reading dropped to 0.07–0.08), and day 15 (E, Baumé meter reading stabilized). All the samples were split in half. The metagenomic samples were cryopreserved at −80 °C for subsequent DNA extraction and next-generation sequencing, and the metabolome samples were cryopreserved at −20 °C for metabolite analysis.

### 2.2. Physicochemical Analysis

The total sugar, titratable acidity, pH, ethanol, volatile acid, and yeast assimilated nitrogen (YAN) were detected using an FTIR Wine Analyzer (Lyza 5000 Wine, Graz, Austria).

### 2.3. DNA Extraction and Metagenomic Sequencing

Genomic DNA was extracted as per the protocols for EZNA^®^ DNA Kits (Omega Bio-Tek, Norcross, GA, USA). DNA quality was detected using the Qubit and NanoDrop system (both Thermo Fisher Scientific, Waltham, MA, USA). The next step was the construction of a DNA library (insert size 350 bp) via the NEBNext^®^ ΜLtraTMDNA Library Prep Kit (Illumina, NEB, San Diego, CA, USA) and its sequencing on the Illumina NovaSeq 6000 platform (Illumina Inc., San Diego, CA, USA) at Gene Denovo Co., Ltd. (Guangzhou, China). DIAMOND 0.9.24 was employed for functional annotation, where the unigene sequences were aligned to deposited ones in such protein databases as the Kyoto Encyclopedia of Genes and Genomes (KEGG) and the National Center for Biotechnology Information’s (NCBI) non-redundant protein (Nr) database. The taxonomic profile was generated using clean reads via Kaiju 1.6.3. Species abundance is the ratio of the number of reads for the species to the total number of reads in the sample.

### 2.4. Metabolomics Analysis of UHPLC–MS/MS

The Vanquish UHPLC system and Orbitrap Q Exactive TM HF-X MS (both Thermo Fisher, Maharashtra, Germany) were applied to perform UHPLC–MS/MS analysis. A 17 min linear gradient was adopted to inject the samples onto a 1.9 μm Hypersil Gold column (100 × 2.1 mm), by setting the flow rate to 0.2 mL/min. Eluent A (0.1% aqueous FA) and eluent B (methanol) were utilized in the positive polarity mode, whereas eluent A (pH 9.0, 5 mM ammonium acetate) and eluent B (methanol) were used in the negative polarity mode. The solvent gradient setting was 2% B, 1.5 min; 2–100% B, 12.0 min; 100% B, 14.0 min; 100–2% B, 14.1 min; 2% B, 17 min. The Q Exactive TM HF-X MS was run in alternating polarity mode, at a 320 °C capillary temperature, a 3.2 kV spray voltage, a 40 arb flow rate of sheath gas, and a 10 arb aux gas flow rate.

Compound Discoverer 3.1 (CD3.1, Thermo Fisher) was applied to process the raw UHPLC–MS/MS files, thereby performing alignment, picking, and quantitation of the peaks for every metabolite. The major parameter settings were a 0.2 min retention time tolerance; a 5 ppm actual mass tolerance; a 30% signal intensity tolerance; a signal/noise ratio of 3; as well as a minimum intensity of 100,000. The subsequent step was the normalization of the peak intensities into the overall spectral intensity. The molecular formula was forecasted by utilizing normalized data based on the molecular ion peaks, additive ions, and fragment ions. Finally, the peaks were matched with the mzVault, mzCloud, and Mass List database to derive the precise qualitative and relative quantitative outcomes.

### 2.5. Data Analysis

Multivariate statistical analysis of the distance matrix, including the PCA and PCoA, was performed through the “labdsv” package. The associations of the microbial populations with non-volatile metabolites were unraveled by redundancy analysis (RDA) through the “vegan” package. An alluvial map elucidates the dynamics and succession of dominant microbial taxa during fermentation through the “ggalluvial” package. The partial least squares discriminant analysis (PLS-DA) model in SIMCA 14.1 (UMETRICS, Umeaa, Sweden) was employed for identifying the differential metabolites during fermentation. The ROC curve was used to evaluate the ability of the differential metabolites to differentiate the samples during fermentation. Differences in the distribution of certain data were analyzed by ANOVA and PERMANOVA tests, using the “vegan” package. Significant inter-group differences were indicated by different letters and regarded as significant at the *p* < 0.05 level.

## 3. Results and Discussion

### 3.1. Dynamics of Physicochemical Factors During Fermentation

**Figure 1:**
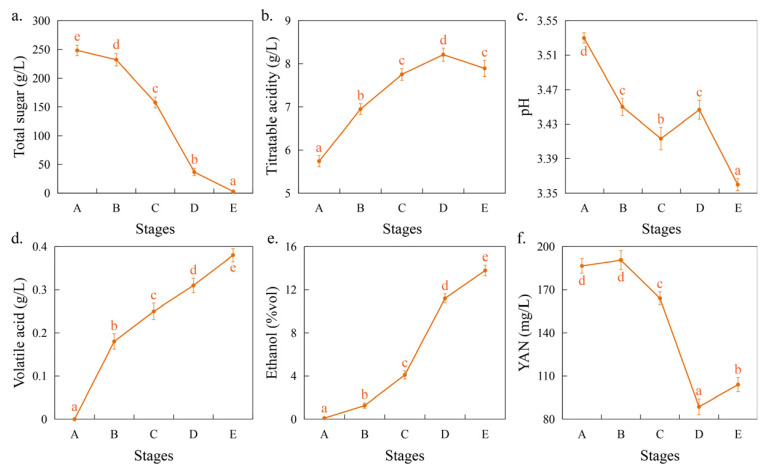
Changes in the physicochemical factors during spontaneous fermentation. Note: (**a**) total sugar; (**b**) titratable acidity; (**c**) pH; (**d**) volatile acid; (**e**) ethanol; (**f**) yeast assimilated nitrogen (YAN). Different letters indicate significant differences between different stages (*p* < 0.05), while the same letters indicate no significant differences.

During fermentation, a total of 15 samples were collected at five time points. Changes in the total sugar, titratable acidity, pH, and YAN content during fermentation are often used to determine the fermentation status [15]. During the fermentation process, the total sugar content decreased significantly (*p* < 0.05), from the initial 248.24 g/L to the final 2.88 g/L (Figure 1a), indicating that the indigenous yeasts could complete the alcoholic fermentation process, which was lower than the national standard (GB/T 15037) of 4g/L in China. The titratable acidity increased significantly during fermentation (*p* < 0.05), from the initial 5.74 g/L to the final 7.89 g/L, while the pH decreased significantly (*p* < 0.05), from the initial pH of 3.53 to the final pH of 3.36 (Figure 1b,c). Morata et al. (2019) demonstrated that using grape musts from hot climates as a fermentation substrate and inoculation with the indigenous yeast, *Lachancea thermotolerans*, in combination with *S. cerevisiae* for co-fermentation, can increase the titratable acidity from 4 g/L to 9 g/L and decrease the pH from 3.9 to 3.4 [16], indicating the presence of acidifying yeasts among indigenous microorganisms. The volatile acid increased significantly during fermentation (*p* < 0.05) and was 0.38 g/L at the end of fermentation (Figure 1d), which was significantly lower than the national standard of 1.2 g/L in China. Ethanol was significantly increased during fermentation (*p* < 0.05) and was 13.8%vol at the end of fermentation (Figure 1e). The YAN decreased first and then increased during fermentation, decreasing to 88.67 mg/L at stage D and increasing to 104 mg/L at stage E (Figure 1f). During fermentation, yeast absorbs and utilizes YAN and other nutrients to support its growth and fermentation activities, thereby leading to a gradual decrease in the YAN concentration in the fermentation medium; later in the fermentation process, yeast cells may begin to autolysate, releasing intracellular sources of nitrogen, such as amino acids and peptides [17].

### 3.2. Structure and Composition of Microbial Community During Fermentation

The microbial community structure and composition of samples from different fermentation stages were analyzed based on metagenomics. DNA from eukaryotic and prokaryotic sources was extracted using Illumina NovaSeq 6000, fragments and sequencing were performed, and 102.49 Gbp original reads in terms of PE150 were obtained, and 102.14 Gbp clean reads were obtained after quality control. The clean reads were then assembled and filtered using MEGAHIT software and a total of 989,711 long sequences of Contigs were obtained. A total of 149,971 unigene sequences were identified.

**Figure 2:**
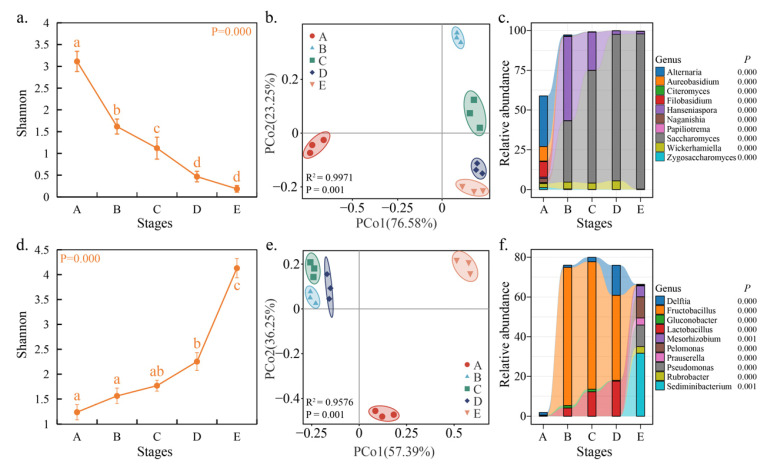
Microbial community diversity and composition during spontaneous fermentation. Note: α-diversity (Shannon index) of fungi (**a**) and bacteria (**d**) based on species level during fermentation; PCoA showed the distribution pattern of fungi (**b**) and bacteria (**e**) communities during fermentation; relative abundance changes in dominant fungi (**c**) and bacteria (**f**) taxa at the genus level (top 10).

For fungi, the Shannon index-based species-level diversity was significantly decreased during fermentation (Figure 2a, ANOVA, *p* < 0.05). The PCoA showed that spontaneous fermentation processes were grouped according to their fermentation stage, with the first two principal axis components explaining 99.83% of the total variance (Figure 2b), indicating differences in the fungal community structure at different fermentation stages. The PERMANOVA also confirmed significant differences in the fungal communities between different fermentation stages (R^2^ = 0.9971, *p* = 0.001). After entering the fermentation peak period (C to E), the distance between C, D, and E samples, based on the Bray–Curtis distance, gradually shortened, indicating that the differences between the fungal communities gradually decreased as the fermentation process progressed (Figure 2b). Tracking the dominant taxa (top 10 in terms of relative abundance) can reveal the dynamics and succession of fungal communities during fermentation. The stacked graph shows significant differences in the relative abundance of the dominant taxa during fermentation (Figure 2c, ANOVA, *p* < 0.05). At stage A, abundant yeast taxa were detected, of which *Saccharomyces* accounted for 0.13% of the community, and non-*Saccharomyces* yeasts, namely *Hanseniaspora*, *Wickerhamiella*, *Filobasidium*, *Naganishia*, *Zygosaccharomyces*, and *Papiliotrema* accounted for 17.76% of the community; in addition, an abundance of Alternaria species were detected, accounting for 31.80% of the community (Figure 2c). Non-*Saccharomyces* yeasts typically exhibit weaker fermentative power during alcohol fermentation, but they can produce a diverse range of flavor compounds through various metabolic pathways, which can increase the aroma complexity and improve the quality of wine [9]. After fermentation had started, the relative abundance of *Saccharomyces* gradually increased, that of *Hanseniaspora* and *Wickerhamiella* first increased and then decreased, and that of other taxa gradually decreased, such as *Alternaria*, *Filobasidium*, and *Aureobasidium* (Figure 2c). Spontaneous wine fermentation is triggered by indigenous microorganisms, particularly non-*Saccharomyces* yeasts like *Candida*, *Hanseniaspora*, and *Pichia*, which are typically present in the initial stages of wine fermentation [4]. Due to the high ethanol content, low oxygen level, and nutrient consumption, the fermentation environment became harsh and non-*Saccharomyces* yeasts were gradually replaced by more fermentatively robust yeast (such as *Kluyveromyces* and *Torulaspora*); finally, *S. cerevisiae* dominated the fermentation process, became the dominant strain, and completed the alcoholic fermentation [18], which was the main reason for the decrease in fungal diversity during fermentation. At the end of fermentation (stage E), *Saccharomyces* accounted for 97.53% of the relative abundance, while *Hanseniaspora* constituted 1.62%, with other taxa present at levels below 0.1% (Figure 2c). Studies have demonstrated that *H. uvarum* exhibits a consistently high fermentation capacity, maintaining viability even at ethanol concentrations of up to 12% (*v*/*v*) [19].

For bacteria, the Shannon index-based species-level diversity significantly increased during fermentation (Figure 2d, ANOVA, *p* < 0.05). The most likely explanation for this phenomenon is that certain yeasts can produce varying amounts of inhibitory or stimulatory compounds, according to which bacteria are more sensitive [20,21,22]. However, the mechanism underlying this interaction is not fully understood. The PCoA showed that spontaneous fermentation processes are grouped according to their fermentation stage, with the first two principal axis components explaining 93.64% of the total variance (Figure 2e), indicating differences in the bacterial community structure at different fermentation stages. The PERMANOVA also confirmed significant differences in the bacterial communities between different fermentation stages (R^2^ = 0.9576, *p* = 0.001). Based on the weighted UniFrac distance, the distance between samples B, C, and D was smaller compared with samples A and E, suggesting that the bacterial communities’ dissimilarity was greater before and after fermentation, while less pronounced during fermentation (Figure 2e). The stacked graph shows significant differences in the relative abundance of the dominant taxa (top 10) during the fermentation process (Figure 2f, ANOVA, *p* < 0.05). During fermentation, abundant lactic acid bacteria, such as *Fructobacillus* and *Lactobacillus*, were detected, but their relative abundance was significantly reduced at the end of fermentation (Figure 2f). Some yeasts have the ability to inhibit the growth of lactic acid bacteria. For example, *S. cerevisiae* can produce sulfites via the sulfate reduction pathway, which, in acidic conditions, form molecular SO_2_. This molecule SO_2_ possesses higher antibacterial activity and can inhibit the growth of lactic acid bacteria [23]. During fermentation, an abundance of the *Gluconobacter* genus were also detected, with their relative abundance initially increasing and then decreasing (Figure 2f). The bacteria are known to produce volatile acids by metabolizing glucose [24], which significantly impacts the flavor and quality of wine. During fermentation, *Delftia* was also detected in abundance, particularly at stage D, where its relative abundance reached 15.11% (Figure 2f). *Delftia* species form symbiotic relationships with grapevines, affecting grape growth and development and, consequently, impacting the quality of wine [25]. It is noteworthy that *Sediminibacterium*, *Pelomonas*, *Prauserella*, and *Rubrobacter* were only detectable at the end (E stage) of the fermentation process (Figure 2f). These taxa may influence wine quality by producing organic acids or competing with yeasts for nutrients [9]. Additionally, an abundance of *Pseudomonas* and *Mesorhizobium* were also detected at the end of fermentation (stage E) (Figure 2f). These genera are integral components of the vineyard soil microbiome, with some species capable of promoting plant growth or inhibiting pathogenic bacteria, and they provide nitrogen sources required for plants through nitrogen fixation [25].

### 3.3. KEGG Functional Classifications of Microbial Communities During Fermentation

**Figure 3:**
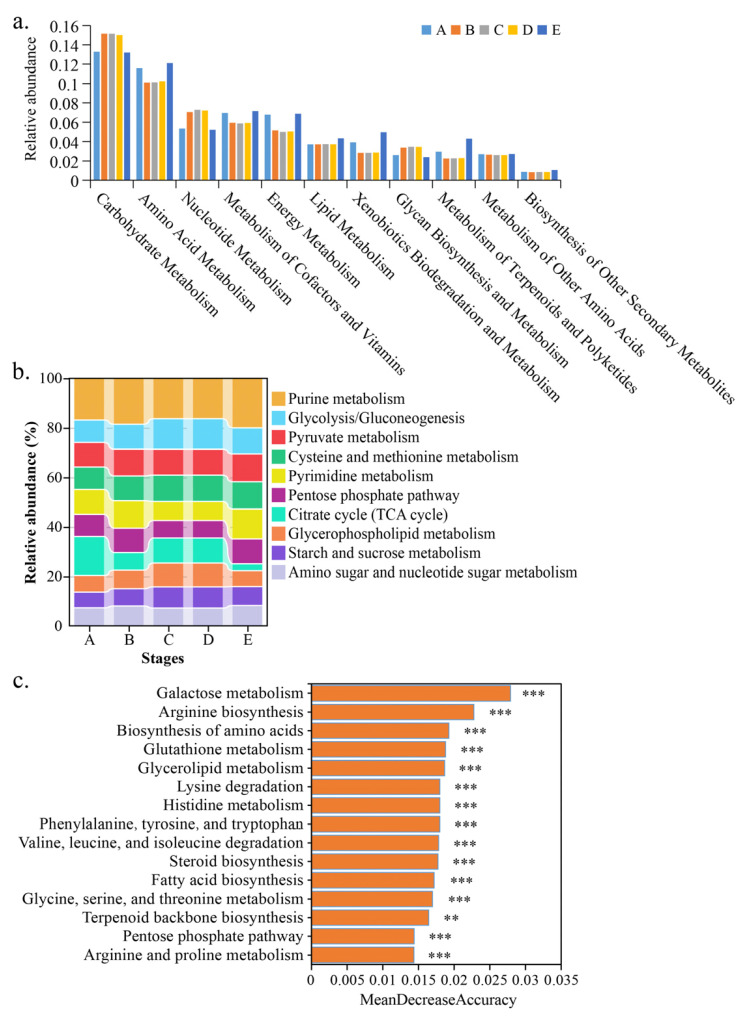
KEGG function classification of microbial community during fermentation. Note: pathways related to metabolism at level B (**a**) and level C (**b**) during fermentation, respectively; the importance of C-level pathways related to metabolism (**c**) was ranked using random forest. Significance, ** *p* < 0.01, *** *p* < 0.001.

To gain a better understanding of the metabolic pathways associated with the identified genes, the genes were mapped to the KEGG pathway database. A total of 412 C-level metabolic pathways were identified in the annotated dataset. In the pathway related to metabolism at level B, the identified genes are mainly related to amino acid metabolism, carbohydrate metabolism, nucleotide metabolism, and metabolism of co-factors and co-factor vitamins, energy metabolism, lipid metabolism, xenobiotics biodegradation and metabolism, glycan biosynthesis and metabolism, metabolism of terpenoids and polyketides, metabolism of other amino acids, and the biosynthesis of other secondary metabolites (Figure 3a). Amino acids are not only essential nutrients for the growth and fermentation of *S. cerevisiae*, but they are precursors to many aromatic substances in wine [15]; carbohydrate metabolism involves yeast interacting with the sugars in grape juice, converting them into alcohol and carbon dioxide [9]; lipid metabolism plays a crucial role in maintaining the integrity and function of yeast membranes, which in turn influences the production of key aroma compounds, such as esters and higher alcohols [3]; in terpenoid metabolism, yeast can produce terpenoid compounds with aroma activity, by metabolizing the terpenoid precursor substances in grapes [9]; other metabolic pathways mainly affect the growth and reproduction of yeast, but by regulating these metabolic pathways, the quality of wine can be improved. Among the metabolism-related pathways, purine metabolism, glycolysis/gluconeogenesis, pyruvate metabolism, cysteine and methionine metabolism, pyrimidine metabolism, the pentose phosphate pathway, the citrate cycle, glycerophospholipid metabolism, starch and sucrose metabolism, amino sugar and nucleotide sugar metabolism at level C were particularly abundant during fermentation (Figure 3b). The random forest method was used to rank the importance of the metabolism-related pathways. The MeanDecreaseAccuracy index showed that galactose metabolism, arginine biosynthesis, the biosynthesis of amino acids, glutathione metabolism, glycerolipid metabolism, lysine degradation, histidine metabolism, phenylalanine tyrosine and tryptophan biosynthesis, valine leucine and isoleucine degradation, steroid biosynthesis, fatty acid biosynthesis, glycine serine and threonine metabolism, terpenoid backbone biosynthesis, the pentose phosphate pathway, and arginine and proline metabolism at level C were the 15 most important metabolic pathways during fermentation (Figure 3c).

### 3.4. Evolution of Metabolic Profile During Spontaneous Fermentation

**Figure 4:**
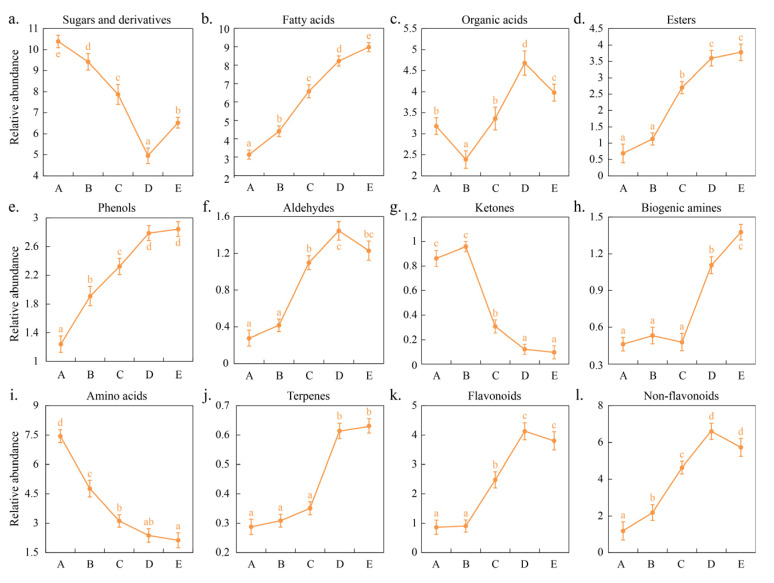
The change in total content of various flavor compounds during fermentation. Note: different letters indicate significant differences between different stages (*p* < 0.05), while the same letters indicate no significant differences. (**a**–**l**) respectively represent sugars and derivatives, fatty acids, organic acids, esters, phenols, aldehydes, ketones, biogenic amines, amino acids, terpenes, flavonoids, and non-flavonoids.

UHPLC–MS/MS was used to evaluate the metabolic profiles at different stages of spontaneous fermentation of wine to better understand the changes in flavor compounds. Molecules in wine include a large number of primary and secondary metabolites. In this study, important metabolites during fermentation were evaluated, among which 19 sugars and derivatives, 20 fatty acids, 10 organic acids, 5 esters, 4 phenols, 3 aldehydes, 1 ketone, 5 biogenic amines, 17 amino acids, 1 terpene, 12 flavonoids, and 17 non-flavonoids were identified (Supplementary Table S1). Figure 4 shows the variation trend in the total content of these flavor families during fermentation. The total content of sugars and derivatives decreased from 9.93% (A) to 5.05% (C) and then increased to 7.58% (E) during fermentation (Figure 4a). Glucose and fructose, as the primary sugar components, are consumed by yeast during alcoholic fermentation to produce ethanol and carbon dioxide; in the later stages of fermentation, polysaccharides with lower molecular weights in grape skins, such as arabinogalactan proteins and rhamnogalacturonan II, are gradually released and increase with the extension of the maceration time; furthermore, due to yeast autolysis and the action of β-glucanases, mannoproteins and glucans are also released in large quantities [26]. These sugars enhance the sweetness of wine and contribute to the fullness of wine [11]. The total content of fatty acids, esters, phenols, and aldehydes gradually increased during fermentation and were 8.98%, 3.78%, 2.84%, and 1.23% at the end of fermentation (E), respectively (Figure 4b,d–f). Studies have shown that the concentration of fatty acids, esters, and aldehyde compounds gradually increased as the fermentation process progressed [18], which was mainly affected by *S. cerevisiae*, non-*Saccharomyces* yeasts, and lactic acid bacteria [27]. The low concentration of volatile fatty acids can give the wine a fruity, cheesy, and fatty taste; ester compounds can give wine a pleasant smell, such as a fruity and floral smell; aldehydes provide the characteristic aromas of wine, such as green, nut, and toast aromas [13]. These compounds can enhance the complexity of the aroma of wine. The total organic acid content decreased from 3.18% (A) to 2.39% (B), then increased to 4.68% (D), and finally decreased to 3.98% (E) (Figure 4c). During wine fermentation, the content of tartaric acid, malic acid, ascorbic acid, and citric acid typically decrease; oxalic acid and succinic acid increase first and then decrease; and lactic acid and acetic acid increase at the end of fermentation [28,29]. The variety and concentration of organic acids dictate the acidity and pH levels of wine. These acids exhibit antimicrobial effects, and a lower pH is conducive to maintaining the wine’s freshness [26]. The total ketone content increased from 0.86% (A) to 0.96% (B) and then gradually decreased to 0.09% (E) (Figure 4g). Tang et al. (2021) investigated the changes in metabolites during the fermentation of Vidal ice wine and the results showed that the total content of ketones gradually decreased as the fermentation progressed [30]. Ketones can give wine a pleasant aroma, such as fruity and floral aromas; for example, 6-methyl-5-hepten-2-one enhances the citrus aroma of wine [25]. The total content of biogenic amines remained relatively unchanged during the early stages of fermentation (A to C), but gradually increased to 1.38% (E) during the later stages (Figure 4h); the total content of amino acids gradually decreased to 2.12% (E) during fermentation (Figure 4i). During wine fermentation, amino acid decarboxylase can catalyze the corresponding free amino acids to decarboxylate and form the corresponding biological amine compounds [31]. However, conflicting conclusions have been drawn regarding the influence of the amino acid concentration on BA formation [32,33,34]. Amino acids serve as precursors for numerous aromatic compounds in wine. Specific amino acids, such as tryptophan, tyrosine, and phenylalanine, can be converted into volatile phenols, ethers, and esters through the shikimate pathway, which significantly impacts the flavor profile of wine [31]. The total content of terpenes gradually increased to 0.64% (E) during fermentation (Figure 4j). Skin maceration of grapes can increase the content of free and some bound terpenes in wine [35]; in addition, the production of terpene compounds depends on some non-Saccharomyces yeasts, which can secrete β-glucosidase and hydrolyze the glycoside bond to produce terpenes, such as linalsol and geranyl [36]. Specific terpenes, such as monoterpenes and sesquiterpenes, contribute to the fruity and floral aroma characteristics of young wines [33]. The total content of flavonoids and non-flavonoids gradually increased to 4.13% and 6.61% (D) during fermentation, respectively, but decreased slightly at the end of fermentation (E) to 3.49% and 5.73%, respectively (Figure 4k,l). Qin et al. (2023) found that maceration could also promote the extraction of flavonoids from Black Mulberry peel into juice and that the concentration increased with the extension of the maceration time; furthermore, the concentration of various flavonoid compounds exhibit distinct changes during fermentation; the majority, such as myricetin, epicatechin, and rutin, show an increase, whereas a minority, including kaempferol, luteolin-7-O-glucoside, quercetin, and epicatechin, demonstrate a decrease. During the later stages of fermentation, phenolic compounds undergo oxidation reactions or interact with proteins to form complexes that precipitate, both of which contribute to a reduction in the total content of flavonoids [37,38]. Similarly, maceration promoted the release of non-flavonoids from grape skins into the juice/wine during fermentation [39], but later in fermentation, protein interactions with non-flavonoids (e.g., pectin, phenolic acids, tannins) formed a complex precipitate, which also resulted in a decrease in the content of non-flavonoids [40]. Non-flavonoid compounds mainly include phenolic acids, which can have physicochemical interactions with aroma compounds in wine and affect the retention or release of aroma compounds [39].

The PLS-DA model was constructed to identify the differential metabolites during spontaneous fermentation. In the model, the independent variable fitting index (R^2^X) was 0.955, the dependent variable fitting index (R^2^Y) was 0.994, and the model prediction index (Q^2^) was 0.965, indicating that the model was successfully constructed, and the prediction rate of the differential metabolites during fermentation was 96.5% (Figure S1a). The PLS-DA results showed that the samples at different fermentation stages were obviously separated, indicating that the metabolite profiles during fermentation were different (Figure S1a). To avoid the overfitting of the PLS-DA model, 200 cross-validations were conducted to verify that the Q^2^ values were all less than 0, indicating that overfitting of the model had not occurred (Figure S1b). Therefore, the model could identify differential metabolites during the fermentation process. The model generated two principal components in total, among which, dodecanoic acid, histidine, diacetyl, fructose, butyrate, capric acid, mannose and palmitic acid, proline, and trehalose, contributed the most to the changes in the two principal components during fermentation (Figure S1c). In addition, according to the VIP values > 1 and *p* values < 0.05, the PLS-DA model also identified 16 different metabolites, namely fructose, mannose, trehalose, histidine, tyrosine, proline, arginine, palmitic acid, butyrate, diacetyl, galactose, and linoleic acid, palmitoleic acid, dodecanoic acid, capric acid, and caprylic acid. ROC curve analysis was performed to evaluate the potential of these 16 differential metabolites to distinguish the fermentation stage and AUC analysis was performed to test the reliability of the differential metabolites. The results showed that these 16 differential metabolites had high discriminating power in terms of the samples during fermentation (AUC > 0.9, Figure S1d).

### 3.5. Potential Analysis of Key Flavor Compound Formation Mechanisms During Spontaneous Fermentation of Cabernet Sauvignon

**Figure 5:**
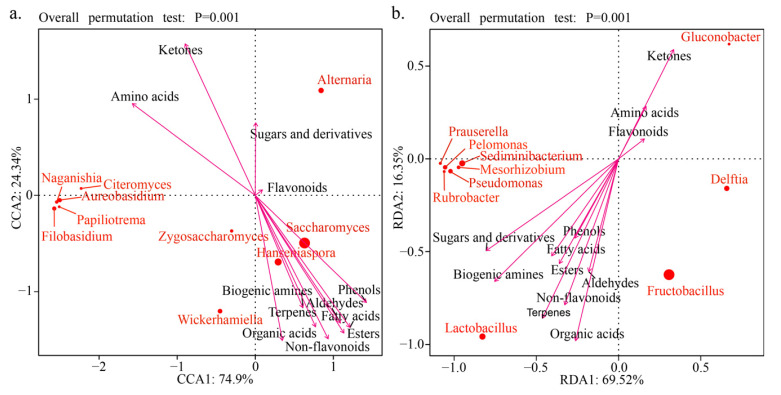
RDA analysis of dominant fungal (**a**) and bacterial (**b**) taxa abundance and flavor metabolite concentration during wine fermentation.

The dominant microbial taxa associated with quantitative traits of wine metabolites were identified using redundancy analysis. The RDA indicated that the relative abundance of the dominant microbial taxa was correlated with metabolite concentrations that are important for wine flavor (e.g., organic acids, fatty acids, esters, phenols, aldehydes, terpenes, and phenols) (Figure 5). For instance, yeasts such as *Zygosaccharomyces*, *Saccharomyces*, *Hanseniaspora*, and *Wickerhamiella* were positively correlated with the primary aroma compounds in wine, including organic acids, fatty acids, esters, aldehydes, phenols, terpenes, flavonoids, and non-flavonoids; bacteria, such as *Lactobacillus* and *Fructobacillus*, were positively correlated with sugars and derivatives, organic acids, fatty acids, esters, aldehydes, phenols, terpenes, and non-flavonoids, and *Gluconobacter* was positively correlated with ketones, amino acids, and flavonoids. These microorganisms may directly or indirectly regulate the chemical composition of wine through their metabolic activities, thereby affecting the flavor and quality of wine. For example, terpenoid compounds, which are derivatives of grapes, can be modified by yeasts and lactic acid bacteria that secrete glycosidases during fermentation, thereby influencing the flavor and aroma of wine [41]; ethyl ester and acetic ester are important ester compounds in wine, contributing importantly to the fruity and floral aroma characteristics of wine; for example, ethyl butyrate can give wine sweet strawberry, apple, and other fruity aromas, and phenyl ethyl acetate can give wine honey, rose, and lilac aromas. *S. cerevisiae* and non-*Saccharomyces* yeasts (e.g., *Hanseniaspora uvarum*, *Starmerella bacillaris*, *Metschnikowia pulcherrima*) can produce rich ester compounds during fermentation [42].

**Figure 6:**
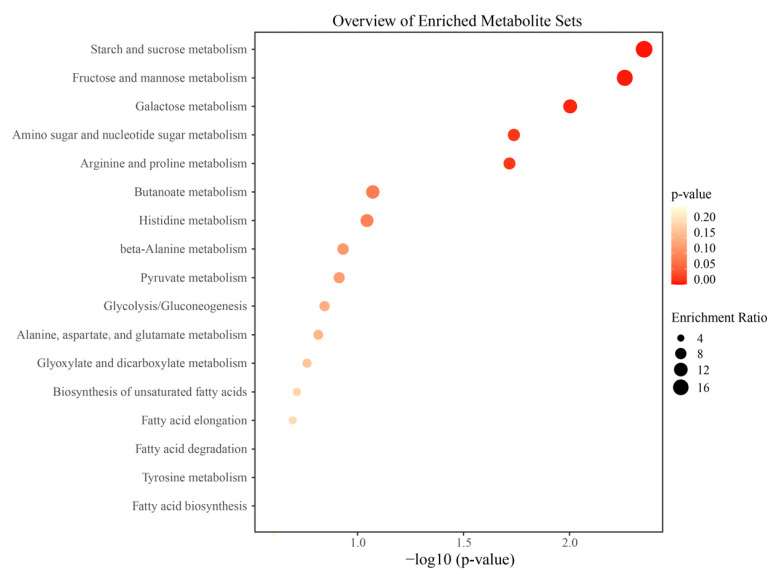
Enrichment analysis of differential metabolites during fermentation.

The enrichment analysis of metabolic pathways can elucidate the metabolic mechanism in spontaneous fermentation. In this study, differential metabolites were used for metabolic pathway enrichment analysis based on the KEGG database (Figure 6). The results showed that a total of 17 metabolic pathways were identified, including starch and sucrose metabolism (ko00500); fructose and mannose metabolism (ko00051); galactose metabolism (ko00052); amino sugar and nucleotide sugar metabolism (ko00520); arginine and proline metabolism (ko00330); butanoate metabolism (ko00650); histidine metabolism (ko00340); β-alanine metabolism (ko00410); pyruvate metabolism (ko00620); glycolysis/gluconeogenesis (ko00010); alanine, aspartate, and glutamate metabolism (ko00250); glyoxylate and dicarboxylate metabolism (ko00630); biosynthesis of unsaturated fatty acids (ko01040); fatty acid elongation (ko00062); fatty acid degradation (ko00071); tyrosine metabolism (ko00350); and fatty acid biosynthesis (ko00061). The above metabolic pathways are involved in the synthesis, decomposition, transformation, and utilization of sugars, amino acids, and fatty acids. These metabolic pathways are of particular interest in this study, as they are potentially involved in the production of flavor compounds within Cabernet Sauvignon wines.

Based on the enrichment analysis results, in conjunction with metagenomic data, a comprehensive analysis of the metabolic pathways involved in the spontaneous fermentation of Cabernet Sauvignon wine is presented. The predicted network diagram of the metabolic pathways involved in flavor formation during fermentation is shown in Figure S2 in the Supplementary Materials. The metagenomic analysis revealed that genes encoding enzymes associated with the aforementioned metabolic networks are primarily attributed to *Saccharomyces*, *Hanseniaspora*, *Zygosaccharomyces*, *Wickerhamiella*, *Lactobacillus*, and *Fructobacillus*. Glycolysis is the primary metabolic pathway according to which yeast converts glucose into energy and is a precursor for biosynthesis. For instance, it provides energy for cells, produces alcohol, and serves as a precursor for the synthesis of flavor compounds, such as esters, higher alcohols, and volatile acids [43]. The primary taxa involved in glycolysis were *Saccharomyces*, *Hanseniaspora*, *Lactobacillus*, and *Fructobacillus*, with the associated enzymes being hexokinase (EC 2.7.1.1), glucokinase (EC 2.7.1.2), phosphohexomutase (EC 5.3.1.9), phosphohexokinase (EC 2.7.1.11), aldolase (EC 4.1.2.13), triosephosphate dehydrogenase (EC 1.2.1.12), enolase (EC 4.2.1.11), and pyruvate synthase (EC 1.2.7.1). Pyruvate serves as a primary precursor for the synthesis of acids, alcohols, and esters [41]. The primary taxa involved in pyruvate metabolism were *Hanseniaspora* and *Zygosaccharomyces*, with the associated enzymes being pyruvate synthase (EC 1.2.7.1), pyruvate oxidase (EC 1.2.3.3), and acetolactate synthase (EC 2.2.1.6). Sucrose, an important sugar in grapes, is hydrolyzed into glucose and fructose as a result of the action of sucrase, which are subsequently fermented by yeast into ethanol and CO_2_; fructose, another type of sugar present in grapes, can be directly utilized by yeast; mannose, present in grapes in lower concentrations, can be utilized by yeast to produce glycerol and ethanol; galactose is not a primary sugar source, but it can be utilized by yeast, which is converted into galactose-1-phosphate by galactokinase, subsequently entering the glycolytic pathway; amino sugars (e.g., glucosamine) and nucleotide sugars (e.g., ribose) are integral components in cellular metabolism and anabolic pathways, which yeast can exploit to synthesize cell wall components, generate energy, and produce biosynthetic precursors [44]. The primary taxa involved in sugar decomposition, conversion, and utilization were *Saccharomyces*, *Hanseniaspora*, *Zygosaccharomyces*, *Wickerhamiella*, and *Lactobacillus*, with the associated enzymes being phosphohexokinase (EC 2.7.1.11), aldolase (EC 4.1.2.13), triosephosphate dehydrogenase (EC 1.2.1.12), enolase (EC 4.2.1.11), phosphohexomutase (EC 5.3.1.9), and glucokinase (EC: 2.7.1.2). In wine fermentation, amino acids have a significant impact on both yeast growth and the aroma profile of the wine. For instance, arginine is converted into proline through the arginine decarboxylase pathway (AGD pathway), which plays a crucial role in yeast growth and metabolic regulation; the metabolism of proline contributes to the maintenance of the cellular redox balance and osmotic pressure balance; histidine metabolism may influence the utilization of other nitrogen sources by yeast; alanine serves not only as a nitrogen source, but also participates in energy metabolism and the tricarboxylic acid (TCA) cycle during yeast fermentation; aspartate and glutamate are key molecules in nitrogen and carbon metabolism within yeast cells, participating in the synthesis of various biomolecules, including nucleic acids, proteins, and intermediates of energy metabolism; tyrosine is a precursor to the synthesis of phenolic compounds, which significantly influence the color and flavor of wine [45]. The primary taxa involved in the synthesis, degradation, transformation, and utilization of amino acids were *Saccharomyces*, *Hanseniaspora*, *Zygosaccharomyces*, *Lactobacillus*, and *Fructobacillus*, with the associated enzymes being aspartate transaminase (EC 2.6.1.1), argininosuccinate synthase (EC 6.3.4.5), aspartate carbamoyltransferase (EC 2.1.3.2), aldehyde dehydrogenase (EC 1.2.1.3), beta-ureidopropionase (EC 3.5.1.6), histidinol dehydrogenase (EC 1.1.1.23), carnosine N-methyltransferase (EC 2.1.1.22), dihydropyrimidinase (EC 3.5.2.2), and 4-hydroxyphenylpyruvate dioxygenase (EC 1.13.11.27). Metabolic pathways associated with fatty acids also influence the flavor, aroma, and mouthfeel of wine. In wine fermentation, butyrate metabolism primarily involves the synthesis and degradation of butyric acid and its derivatives; polyunsaturated fatty acids, e.g., oleic acid, arachidonic acid, docosahexaenoic acid, and eicosapentaenoic acid, are essential lipid molecules involved in the constitution of biological membranes, signal transduction, and energy storage; the oxidation of fatty acids can provide an additional energy source, particularly in conditions where there is limited carbohydrate availability; the biosynthesis of fatty acids is crucial for cell growth and the maintenance of cellular membrane integrity [46]. The primary taxa involved in the synthesis, degradation, transformation, and utilization of fatty acids were *Saccharomyces*, *Hanseniaspora*, *Zygosaccharomyces*, *Wickerhamiella*, *Lactobacillus*, and *Fructobacillus*, with the associated enzymes being acetyl-CoA carboxylase (EC 6.4.1.2), [acyl-carrier protein] S-malonyl transferase (EC 2.3.1.39), yeast fatty acid synthase (EC 2.3.1.86), β-ketoacyl-[acyl-carrier protein] synthase II (EC 2.3.1.179), 3-oxoacyl-[acyl-carrier protein] reductase (EC 1.1.1.100), and trans-2-enoyl-CoA reductase (EC 1.3.1.38). During fermentation, the concentration of esters, amino acids, organic acids, ketones, higher alcohols, and fatty acids gradually increase, providing the final fermentation products with a rich floral, fruity, herbaceous, and other unique odors. *Saccharomyces*, *Hanseniaspora*, *Zygosaccharomyces*, *Wickerhamiella*, *Lactobacillus*, and *Fructobacillus* may actively participate in the synthesis, degradation, transformation, and utilization of sugars, amino acids, and fatty acids, resulting in higher levels of metabolites.

## 4. Conclusions

This study explores the evolution of flavor metabolites during the spontaneous fermentation of Cabernet Sauvignon wines, as well as the structure and function of the associated microbial communities. During fermentation, the fungal diversity significantly increased, while the bacterial diversity significantly decreased. The total content of fatty acids, organic acids, esters, phenols, aldehydes, terpenoids, flavonoids, and non-flavonoids increased during fermentation, contributing to the rich and distinctive flavor profile of the wine. Metabolic pathways related to the synthesis, degradation, transformation, and utilization of sugars, amino acids, and fatty acids were involved in the formation of key flavor compounds. *Saccharomyces*, *Hanseniaspora*, *Zygosaccharomyces*, *Wickerhamiella*, *Lactobacillus*, and *Fructobacillus* were the dominant taxa during fermentation and were important contributors to the function related to the formation of key flavor compounds in sugar, amino acid, and fatty acid metabolic pathways. Therefore, this study investigated the impact of the indigenous microbiota on key flavor compounds in wine and their formation processes, and provides new insights for the selection of appropriate strains. These strains can be used to construct synthetic microbial communities and serve as starter cultures to regulate the flavor profile of wine.

## Data Availability

The original contributions presented in the study are included in the article/Supplementary Material, further inquiries can be directed to the corresponding author.

## Supplementary Materials

The following supporting information can be downloaded at: https://www.mdpi.com/article/10.3390/foods14020235/s1, Table S1: Metabolite profile of Cabernet Sauvignon wines during spontaneous fermentation; Figure S1: Analysis of differential metabolites during fermentation; Figure S2: Prediction network diagram of metabolic pathways involved in flavor formation during spontaneous fermentation of Cabernet Sauvignon wines.

## References

[1] Duley G., Ferretti C., Morozova K., Longo E., Imperiale S., Ding Y., Poggesi S., Scampicchio M., Boselli E. (2024). How distinctive are ‘Gewürztraminer’ vineyard terroirs in South Tyrol for wine production? A metabolomics-based approach. J. Agric. Food Res..

[2] Liu Q., Zhao X., Jiang Z., Han X., Peng S., Wang J. (2024). Co-evolutionary dynamics of microbial communities and flavor profiles during natural fermentation of Cabernet Sauvignon and Merlot: A comparative study within a single vineyard. Food Res. Int..

[3] Liu D., Legras J.-L., Zhang P., Chen D., Howell K. (2021). Diversity and dynamics of fungi during spontaneous fermentations and association with unique aroma profiles in wine. Int. J. Food Microbiol..

[4] Wei R., Ding Y., Chen N., Wang L., Gao F., Zhang L., Song R., Liu Y., Li H., Wang H. (2022). Diversity and dynamics of microbial communities during spontaneous fermentation of Cabernet Sauvignon (*Vitis vinifera* L.) from different regions of China and their relationship with the volatile components in the wine. Food Res. Int..

[5] Bagheri B., Bauer F.F., Cardinali G., Setati M.E. (2020). Ecological interactions are a primary driver of population dynamics in wine yeast microbiota during fermentation. Sci. Rep..

[6] Lu Y., Sun F., Wang W., Liu Y., Wang J., Sun J. (2020). Effects of spontaneous fermentation on the microorganisms diversity and volatile compounds during ‘Marselan’ from grape to wine. LWT-Food Sci. Technol..

[7] Li R., Lin M., Guo S., Yang S., Han X., Ren M., Song Y., Du L., You Y., Zhan J. (2021). A fundamental landscape of fungal biogeographical patterns across the main Chinese wine-producing regions and the dominating shaping factors. Food Res. Int..

[8] Wei R., Ding Y., Gao F., Zhang L., Wang L., Li H., Wang H. (2022). Community succession of the grape epidermis microbes of cabernet sauvignon (*Vitis vinifera* L.) from different regions in China during fruit development. Int. J. Food Microbiol..

[9] Wei R., Wang L., Ding Y., Zhang L., Gao F., Chen N., Song Y., Li H., Wang H. (2023). Natural and sustainable wine: A review. Crit. Rev. Food Sci. Nutr..

[10] HanyAbdo R.C., MarioVentura D.P., HervéAlexandre M., Rousseaux S. (2020). The establishment of a fungal consortium in a new winery. Sci. Rep..

[11] Welke J.E. (2019). Fungal and mycotoxin problems in grape juice and wine industries. Curr. Opin. Food Sci..

[12] Vyviurska O., Špánik I. (2020). Assessment of Tokaj varietal wines with comprehensive two-dimensional gas chromatography coupled to high resolution mass spectrometry. Microchem. J..

[13] Magyar I., Soós J. (2016). Botrytized wines—Current perspectives. Int. J. Wine Res..

[14] Beltran G., Torija M.J., Novo M., Ferrer N., Poblet M., Guillamón J.M., Rozès N., Mas A. (2002). Analysis of yeast populations during alcoholic fermentation: A six year follow-up study. Syst. Appl. Microbiol..

[15] Zhao C., Su W., Mu Y., Jiang L., Mu Y. (2020). Correlations between microbiota with physicochemical properties and volatile flavor components in black glutinous rice wine fermentation. Food Res. Int..

[16] Morata A., Bañuelos M.A., Vaquero C., Loira I., Cuerda R., Palomero F., González C., SuárezLepe J.A., Wang J., Han S. (2019). *Lachancea thermotolerans* as a tool to improve pH in red wines from warm regions. Eur. Food Res. Technol..

[17] Su Y., Heras J., Beltran G., Gamero A., Querol A., Guillamón J. (2021). Impact of Nitrogen Addition on Wine Fermentation by S. cerevisiae Strains with Different Nitrogen Requirements. J. Agric. Food Chem..

[18] Wei R., Chen N., Ding Y.-t., Wang L., Liu Y.-h., Gao F.-f., Zhang L., Li H., Wang H. (2022). Correlations between microbiota with physicochemical properties and volatile compounds during the spontaneous fermentation of Cabernet Sauvignon (*Vitis vinifera* L.) wine. LWT.

[19] Tristezza M., Tufariello M., Capozzi V., Spano G., Mita G., Grieco F. (2016). The Oenological Potential of *Hanseniaspora uvarum* in Simultaneous and Sequential Co-fermentation with *Saccharomyces cerevisiae* for Industrial Wine Production. Front. Microbiol..

[20] Osborne J.P., Edwards C.G. (2006). Inhibition of malolactic fermentation by *Saccharomyces* during alcoholic fermentation under low- and high-nitrogen conditions: A study in synthetic media. Aust. J. Grape Wine Res..

[21] Comitini F., Ferretti R., Clementi F., Mannazzu I., Ciani M. (2005). Interactions between *Saccharomyces cerevisiae* and malolactic bacter. preliminary characterization of a yeast proteinaceous compound(s) active against *Oenococcus oeni*. J. Appl. Microbiol..

[22] Guilloux-Benatier M., Remize F., Gal L., Guzzo J., Alexandre H. (2006). Effects of yeast proteolytic activity on *Oenococcus oeni* and malolactic fermentation. FEMS Microbiol. Lett..

[23] Zara G., Nardi T. (2021). Yeast Metabolism and Its Exploitation in Emerging Winemaking Trends: From Sulfite Tolerance to Sulfite Reduction. Fermentation.

[24] Silva G.A.R.d., Oliveira S.S.d.S., Lima S.F., Nascimento R.P.d., Baptista A.R.d.S., Fiaux S.B. (2022). The industrial versatility of *Gluconobacter oxydans*: Current applications and future perspectives. World J. Microbiol. Biotechnol..

[25] Bettenfeld P., Canals J.C.i., Jacquens L., Fernandez O., Fontaine F., Schaik E.v., Courty P.-E., Trouvelot S. (2022). The microbiota of the grapevine holobiont: A key component of plant health. J. Adv. Res..

[26] Jones-Moore H.R., Jelley R.E., Marangon M., Fedrizzi B. (2021). The polysaccharides of winemaking: From grape to wine. Trends Food Sci. Technol..

[27] Liu S., Lou Y., Li Y., Zhao Y., Laaksonen O., Li P., Zhang J., Battino M., Yang B., Gu Q. (2023). Aroma characteristics of volatile compounds brought by variations in microbes in winemaking. Food Chem..

[28] Tsegay Z.T. (2020). Total titratable acidity and organic acids of wines produced from cactus pear (Opuntia-ficus-indica) fruit and *Lantana camara* (*L. Camara*) fruit blended fermentation process employed response surface optimization. Food Sci. Nutr..

[29] Yu W., Wei Y., Long F., Zhao S., Xiao Y., Gao H. (2024). Insights into the dynamic evolution of quality and sensory attributes in red aril wine during fermentation. LWT.

[30] Tang K., Zhang X., Li J., Jiang W., Xu Y. (2021). Dynamic changes of aroma during Vidal ice wine fermentation. Food Ferment. Ind..

[31] Macoviciuc S., Niculaua M., Nechita C.-B., Cioroiu B.-I., Cotea V.V. (2024). The Correlation between Amino Acids and Biogenic Amines in Wines without Added Sulfur Dioxide. Fermentation.

[32] Lorenzo C., Bordiga M., Pérez-Álvarez E.P., Travaglia F., Arlorio M., Salinas M.R., Coïsson J.D., Garde-Cerdán T. (2017). The impacts of temperature, alcoholic degree and amino acids content on biogenic amines and their precursor amino acids content in red wine. Food Res. Int..

[33] Belda I., Navascués E., Marquina D., Santos A., Calderón F., Benito S. (2016). Outlining the influence of non-conventional yeasts in wine ageing over lees. Yeast.

[34] Martınez-Pinilla O., Guadalupe Z., Hernandez Z., Ayestaran B. (2013). Amino acids and biogenic amines in red varietal wines: The role of grape variety, malolactic fermentation and vintage. Eur. Food Res. Technol..

[35] Rodrıguez-Bencomo J.J., Mendez-Siverio J.J., Perez-Trujillo J.P., Cacho J. (2008). Effect of skin contact on bound aroma and free volatiles of Listan blanco wine. Food Chem..

[36] Wang X., Fan G., Peng Y., Xu N., Xie Y., Zhou H., Liang H., Zhan J., Huang W., You Y. (2023). Mechanisms and effects of non-*Saccharomyces* yeast fermentation on the aromatic profile of wine. J. Food Compos. Anal..

[37] Qin Y., Xu H., Chen Y., Lei J., Sun J., Zhao Y., Lian W., Zhang M. (2023). Metabolomics-Based Analyses of Dynamic Changes in Flavonoid Profiles in the Black Mulberry Winemaking Process. Foods.

[38] Ozdal T., Capanoglu E., Altay F. (2013). A review on protein–phenolic interactions and associated changes. Food Res. Int..

[39] Radeka S., Orbanic F., Rossi S., Bestulic E., Horvat I., Peršurić A.S.I., Lukic I., Plavša T., Bubola M., Jeromel A. (2024). Evaluating the Impact of Pre-Fermentative and Post-Fermentative Vinification Technologies on Bioactive Compounds and Antioxidant Activity of Teran Red Wine By-Products. Foods.

[40] Jones-Moore H.R., Jelley R.E., Marangon M., Fedrizzi B. (2022). The interactions of wine polysaccharides with aroma compounds, tannins, and proteins, and their importance to winemaking. Food Hydrocoll..

[41] Swiegers J.H., Bartowsky E.J., Henschke P.A., Pretorius I.S. (2005). Yeast and bacterial modulation of wine aroma and flavour. Aust. J. Grape Wine Res..

[42] Belda I., Ruiz J., Esteban-Fernández A., Navascués E., Marquina D., Santos A., Moreno-Arribas M.V. (2017). Microbial Contribution to Wine Aroma and Its Intended Use for Wine Quality Improvement. Molecules.

[43] Waterhouse A.L., Sacks G.L., Jeffery D.W. (2016). Glycolysis. Understanding Wine Chemistry.

[44] Waterhouse A.L., Sacks G.L., Jeffery D.W. (2016). Carbohydrates. Understanding Wine Chemistry.

[45] Waterhouse A.L., Sacks G.L., Jeffery D.W. (2016). Amino Acid Metabolism. Understanding Wine Chemistry.

[46] Waterhouse A.L., Sacks G.L., Jeffery D.W. (2016). Fatty Acid Metabolism. Understanding Wine Chemistry.

