# Supplementary material for: Changes in Microbial Community Diversity and the Formation Mechanism of Flavor Metabolites in Industrial-Scale Spontaneous Fermentation of Cabernet Sauvignon Wines
Source: Foods. 2025 Jan 14;14(2):235. doi: 10.3390/foods14020235 (PMC11764576; doi:10.3390/foods14020235)
Supplement: Supplementary file 1 [file foods-14-00235-s001.zip › foods-3395093-supplementary.pdf]

Supplementary Materials

Table S1. Metabolite profile of Cabernet Sauvignon wine during spontaneous fermentation

Unit: %

Metabolites	CAS	RT	m/z	A	B	C	D	E
Sugars and derivatives								
D-glucose	50-99-7	80.813	178.99767	1.000±0.131	0.995±0.222	0.609±0.346	0.101±0.001	0.001±0.001
D-fructose	57-48-7	301.591	179.0557	0.330±0.066	0.259±0.073	0.268±0.131	0.259±0.103	0.299±0.165
L-Rhamnose	3615-41-6	3.83	228.0851	0.518±0.087	0.658±0.184	0.661±0.335	0.583±0.440	0.927±0.166
D-lactose	63-42-3	6.555	702.26351	0.097±0.041	0.079±0.042	0.061±0.026	0.011±0.065	0.207±0.016
D-turanose	547-25-1	339.596	360.14849	0.618±0.079	0.751±0.304	0.628±0.016	0.233±0.042	0.304±0.046
D-xylose	58-86-6	4.067	323.09325	0.584±0.239	0.561±0.276	0.418±0.202	0.274±0.081	0.305±0.128
Raffinose	512-69-6	7.414	522.20041	0.922±0.089	0.351±0.075	0.373±0.053	0.082±0.009	0.092±0.008
Stachyose	470-55-3	8.202	684.25302	0.351±0.043	0.529±0.024	0.656±0.057	0.373±0.042	0.311±0.049
Trehalose	99-20-7	390.9365	341.1067	0.032±0.038	0.043±0.023	0.0628±0.014	0.069±0.074	0.072±0.107

L-fucose	2438-80-4	4.618	240.98576	0.012±0.012	0.015±0.011	0.013±0.002	0.051±0.164	0.934±0.197
L-arabinose	7296-56-2	3.014	133.04825	0.654±0.073	0.655±0.061	0.558±0.019	0.596±0.054	0.637±0.257
L-arabitol	7643-75-6	1.053	216.15769	1.000±0.247	0.778±0.221	0.499±0.293	0.182±0.175	0.218±0.076
D-mannose	3458-28-4	5.027	198.09634	0.936±0.165	0.693±0.194	0.443±0.153	0.008±0.007	0.010±0.008
D-mannitol	69-65-8	5.032	200.11165	0.402±0.194	0.165±0.241	0.463±0.054	0.488±0.048	0.798±0.059
D-sorbitol	50-70-4	5.799	205.06881	0.880±0.242	0.681±0.276	0.479±0.085	0.212±0.056	0.923±0.246
Myo-inositol	87-89-8	6.543	145.0483	0.769±0.203	0.873±0.077	0.675±0.062	0.137±0.016	0.154±0.132
Glycerol	56-81-5	2.502	223.05938	0.313±0.044	0.464±0.079	0.471±0.130	0.605±0.205	0.522±0.089
D-glucuronic acid	1700908	1.93	389.0851	0.004±0.021	0.010±0.014	0.012±0.002	0.307±0.071	0.481±0.121
Galacturonic acid	9000-69-5	3.648	195.02762	0.977±0.204	0.992±0.211	0.602±0.252	0.337±0.081	0.384±0.122
Fatty acids								
Valeric acid	109-52-4	0.427	227.12463	0.207±0.015	0.314±0.016	0.665±0.063	0.395±0.084	0.626±0.036
Valproic acid	99-66-1	0.73	311.21934	0.105±0.221	0.203±0.045	0.097±0.030	0.347±0.031	0.439±0.073
Caprylic acid	124-07-2	3.061	221.03224	0.302±0.038	0.408±0.093	0.000±0.000	0.713±0.010	0.937±0.013
Suberic acid	505-48-6	1.941	238.1068	0.208±0.015	0.056±0.009	0.184±0.032	0.979±0.032	0.790±0.369

Capric acid	334-48-5	0.722	195.13683	0.044±0.006	0.063±0.007	0.813±0.246	0.182±0.083	0.000±0.000
Sebacic acid	111-20-6	5.378	317.00164	0.056±0.007	0.012±0.002	0.440±0.028	0.039±0.018	0.089±0.016
Pelargonic acid	112-05-0	3.581	158.12772	0.205±0.004	0.162±0.018	0.268±0.161	0.145±0.084	0.261±0.003
Undecanoic acid	112-37-8	0.689	228.19454	0.000±0.000	0.103±0.008	0.849±0.018	0.029±0.001	0.008±0.001
Pentadecanoic acid	1002-84-2	2.989	287.19565	0.457±0.116	0.356±0.102	0.079±0.034	0.977±0.128	0.859±0.015
Stearic acid	21128	2.574	399.13865	0.082±0.019	0.128±0.007	0.001±0.001	0.120±0.003	0.111±0.029
Dodecanoic acid	143-07-7	1.359	218.21029	0.074±0.007	0.037±0.001	0.623±0.043	0.000±0.000	0.344±0.027
Linoleic acid	60-33-3	6.514	395.11384	0.255±0.027	0.419±0.079	0.001±0.001	0.964±0.087	0.665±0.232
Isocaproic acid	646-07-1	0.828	271.1317	0.000±0.000	0.210±0.041	0.266±0.026	0.293±0.062	0.340±0.019
Palmitic acid	21096	2.293	274.27289	0.006±0.004	0.001±0.001	0.948±0.002	0.003±0.001	0.008±0.001
Palmitoleic acid	373-49-9	2.616	277.2151	0.348±0.194	0.325±0.094	0.083±0.030	0.841±0.019	0.499±0.147
Myristic acid	544-63-8	0.73	292.22582	0.281±0.029	0.441±0.022	0.085±0.002	0.580±0.027	0.827±0.173
Myristoleic acid	544-64-9	0.925	244.22588	0.023±0.067	0.160±0.095	0.506±0.089	0.148±0.079	0.117±0.066
3-Hydroxycapric acid	14292-26-3	3.893	188.13816	0.091±0.060	0.077±0.007	0.023±0.006	0.049±0.008	0.222±0.078

3-Hydroxydodecanoic acid	53941-38-1	1.893	258.20525	0.231±0.029	0.549±0.267	0.410±0.001	0.423±0.059	0.906±0.053
16-Hydroxypalmitic acid	506-13-8	2.587	333.2623	0.150±0.045	0.386±0.156	0.240±0.069	1.000±0.080	0.977±0.018
Organic acids								
Citric acid	77-92-9	7.692	193.03317	0.133±0.016	0.640±0.193	0.599±0.018	0.988±0.002	0.255±0.024
Tartaric acid	87-69-4	4.209	192.05483	0.002±0.001	0.554±0.009	0.589±0.115	0.679±0.241	0.675±0.138
L-Malic acid	97-67-6	446.585	133.01368	0.357±0.018	0.158±0.033	0.122±0.042	0.820±0.087	0.173±0.122
Succinic acid	110-15-6	4.306	83.0119	0.989±0.207	0.001±0.001	0.091±0.007	0.089±0.002	0.105±0.003
Citronellic acid	2111-53-7	4.341	170.12729	0.763±0.080	0.286±0.183	0.254±0.047	0.303±0.028	0.380±0.096
Fumaric acid	110-17-8	0.539	250.05843	0.795±0.145	0.003±0.002	0.0001±0.001	0.001±0.001	0.002±0.001
Mesaconic acid	498-24-8	188.371	259.04132	0.064±0.038	0.674±0.022	0.944±0.066	0.819±0.058	0.928±0.135
Salicylic acid	69-72-7	351.503	137.02349	0.048±0.021	0.002±0.001	0.222±0.012	0.469±0.056	0.763±0.066
Shikimic acid	138-59-0	74.5915	173.04444	0.012±0.004	0.008±0.006	0.486±0.006	0.478±0.098	0.669±0.033
Benzoic acid	65-85-0	5.012	123.0429	0.021±0.001	0.064±0.025	0.057±0.008	0.039±0.008	0.034±0.007
Esters								

Butyl lactate	138-22-7	1.773	207.12292	0.063±0.007	0.2544±0.014	0.531±0.065	0.743±0.011	0.826±0.059
Phenyl acetate	122-79-2	1.025	137.05843	0.086±0.008	0.281±0.028	0.062±0.043	0.319±0.073	0.295±0.054
Diethyl sebacate	110-40-7	6.148	373.04893	0.268±0.042	0.499±0.061	0.717±0.123	0.966±0.255	0.859±0.069
Dibutyl sebacate	109-43-3	2.957	353.21557	0.245±0.014	0.058±0.007	0.541±0.251	0.597±0.114	0.798±0.283
4-Hydroxybutanoic [illegible]	96-48-0	6.251	87.04323	0.022±0.007	0.028±0.005	0.847±0.011	0.969±0.022	0.999±0.021
Phenols								
Phenol	108-95-2	37.0335	93.03378	0.233±0.047	0.347±0.036	0.825±0.053	0.889±0.112	0.817±0.068
Resorcinol	108-46-3	2.774	171.06409	0.150±0.013	0.497±0.052	0.222±0.001	0.454±0.058	0.579±0.060
1,3,5-Benzenetriol	108-73-6	5.969	253.06846	0.711±0.124	0.877±0.061	0.709±0.161	0.776±0.036	0.469±0.102
1,2,3-Benzenetriol	87-66-1	6.861	127.03758	0.146±0.026	0.193±0.153	0.568±0.051	0.665±0.109	0.977±0.091
Aldehydes								
Glutaraldehyde	111-30-8	4.101	218.13757	0.231±0.089	0.321±0.103	0.275±0.073	0.499±0.037	0.095±0.093
Hexadecanal	629-80-1	1.406	317.15758	0.007±0.001	0.080±0.030	0.008±0.021	0.043±0.008	0.959±0.002
Phenylacetaldehyde	122-78-1	258.798	103.05309	0.041±0.002	0.014±0.009	0.814±0.015	0.905±0.019	0.168±0.036

Ketone								
Diacetyl	431-03-8	2.401	87.04335	0.863±0.065	0.959±0.040	0.307±0.053	0.122±0.041	0.097±0.054
Biogenic amines								
Histamine	51-45-6	4.368	112.08583	0.031±0.002	0.061±0.021	0.041±0.021	0.072±0.016	0.049±0.001
Spermine	71-44-3	1.098	269.17369	0.157±0.070	0.001±0.001	0.137±0.070	0.618±0.071	0.255±0.086
Tyramine	51-67-2	4.245	120.07965	0.038±0.001	0.067±0.020	0.048±0.001	0.032±0.002	0.028±0.011
Agmatine	-	1.247	130.12156	0.006±0.004	0.094±0.016	0.007±0.001	0.093±0.041	0.756±0.007
Phenylethylamine	64-04-0	10.5	144.07928	0.262±0.014	0.314±0.069	0.252±0.014	0.293±0.004	0.286±0.008
Amino acids								
L-Valine	72-18-4	6.658	118.08499	0.429±0.030	0.234±0.169	0.001±0.001	0.034±0.061	0.021±0.009
L-Tyrosine	60-18-4	2.002	182.07994	0.101±0.002	0.681±0.193	0.149±0.099	0.118±0.014	0.115±0.023
L-Tryptophan	73-22-3	0.526	447.1461	0.979±0.035	0.461±0.096	0.275±0.006	0.129±0.096	0.127±0.090
L-Proline	147-85-3	6.398	269.0849	0.586±0.174	0.637±0.158	0.094±0.001	0.207±0.045	0.276±0.054
L-Lysine	56-87-1	3.868	129.1012	0.264±0.113	0.145±0.025	0.101±0.027	0.122±0.007	0.131±0.076
L-Histidine	71-00-1	8.595	156.0755	0.017±0.004	0.015±0.006	0.011±0.003	0.102±0.057	0.021±0.011

L-Citrulline	372-75-8	6.897	176.1017	0.031±0.007	0.048±0.007	0.049±0.007	0.053±0.028	0.033±0.047
L-Arginine	74-79-3	6.081	175.1177	0.568±0.098	0.159±0.086	0.259±0.099	0.204±0.054	0.126±0.074
D-Ornithine	348-66-3	0.977	303.1501	0.454±0.036	0.451±0.109	0.515±0.014	0.248±0.045	0.141±0.002
DL-Serine	312-84-5	3.097	166.0712	0.320±0.122	0.342±0.121	0.505±0.166	0.040±0.001	0.183±0.099
β-Alanine	107-95-9	3.29	217.0545	0.918±0.093	0.111±0.031	0.117±0.027	0.000±0.000	0.199±0.106
L-Phenylalanine	63-91-2	4.59	166.084	0.444±0.064	0.043±0.065	0.058±0.005	0.259±0.096	0.143±0.034
L-Isoleucine	73-32-5	3.18	132.1008	0.987±0.059	0.101±0.085	0.296±0.052	0.051±0.002	0.299±0.181
4-Aminobutyric acid	20791	7.166	86.05909	0.004±0.001	0.010±0.002	0.003±0.003	0.203±0.058	0.004±0.001
L-Glutamine	5959-95-5	2.988	129.0651	0.458±0.027	0.325±0.026	0.294±0.065	0.218±0.040	0.122±0.042
Asparagine	70-47-3	296.8875	131.0339	0.008±0.003	0.348±0.082	0.399±0.116	0.238±0.005	0.003±0.001
Glutathione	70-18-8	3.935	349.1253	0.859±0.056	0.657±0.022	0.388±0.050	0.107±0.033	0.196±0.097
Terpene								
β-Citronellol	7540-51-4	1.852	201.1262	0.288±0.026	0.309±0.021	0.351±0.021	0.614±0.026	0.637±0.024
Flavonoids								
(-)-Catechin gallate	989-51-5	4.648	459.1001	0.082±0.003	0.077±0.009	0.413±0.092	0.732±0.155	0.392±0.077

(-)-Epicatechin	490-46-0	1.272	273.0749	0.080±0.015	0.078±0.005	0.125±0.004	0.001±0.002	0.193±0.037
(+)-Catechin	154-23-4	6.289	351.1079	0.002±0.001	0.010±0.007	0.034±0.009	0.319±0.008	0.010±0.002
Dihydrokaempferol	480-20-6	100.4205	287.0541	0.069±0.006	0.088±0.013	0.162±0.019	0.227±0.079	0.319±0.009
Epigallocatechin gallate	989-51-5	4.648	459.1001	0.080±0.002	0.099±0.018	0.542±0.060	0.552±0.080	0.634±0.025
Hyperoside	482-36-0	7.704	465.1004	0.098±0.145	0.089±0.001	0.272±0.009	0.110±0.019	0.212±0.016
Isorhamnetin 3-o-glucoside	5041-82-7	154.721	477.0997	0.093±0.006	0.088±0.007	0.617±0.094	0.816±0.074	0.616±0.104
Kaempferol	520-18-3	1.713	287.054	0.084±0.007	0.098±0.002	0.130±0.070	0.889±0.071	0.606±0.045
Procyanidin A1	103883-03-0	7.028	577.1313	0.107±0.054	0.008±0.009	0.053±0.002	0.205±0.078	0.294±0.002
Procyanidin A2	41743-41-3	2.913	637.1535	0.051±0.009	0.066±0.005	0.042±0.030	0.118±0.090	0.238±0.040
Procyanidin B2	29106-49-8	1.733	579.1473	0.089±0.009	0.099±0.004	0.077±0.007	0.029±0.009	0.225±0.010
Procyanidin C1	37064-30-5	254.912	865.1938	0.031±0.006	0.099±0.003	0.003±0.001	0.128±0.007	0.059±0.003
Non-flavonoids								
ϱ-coumaric acid	501-98-4	164.0485	163.0388	0.054±0.006	0.052±0.002	0.347±0.080	0.637±0.135	0.043±0.010
Vanillic acid	121-34-6	1.497	169.0482	0.049±0.003	0.036±0.006	0.165±0.007	0.069±0.046	0.035±0.008
Trans-ferulic acid	537-98-4	151.3455	193.0494	0.050±0.003	0.019±0.013	0.149±0.014	0.398±0.063	0.312±0.032

Trans-3-Coumaric acid	14755-02-3	1.014	147.0427	0.088±0.019	0.090±0.009	0.377±0.092	0.498±0.254	0.091±0.036
Trans-2-Hydroxycinnamic acid	614-60-8	5.001	165.0533	0.079±0.005	0.113±0.004	0.030±0.007	0.895±0.059	0.603±0.054
Syringic acid	530-57-4	174.3525	197.0442	0.099±0.012	0.016±0.009	0.339±0.020	0.399±0.035	0.065±0.002
Caffeic acid	331-39-5	215.113	179.0337	0.083±0.001	0.065±0.051	0.153±0.024	0.169±0.034	0.000±0.000
Gallic acid	149-91-7	5.9	341.0438	0.204±0.042	0.040±0.010	0.088±0.012	0.432±0.042	0.124±0.060
Protocatechuic acid	99-50-3	3.727	172.0592	0.160±0.006	0.288±0.026	0.110±0.019	0.333±0.080	0.725±0.082
Caftaric acid	67879-58-7	348.942	311.0381	0.002±0.003	0.193±0.040	0.032±0.030	0.033±0.018	0.496±0.046
Ethyl gallate	831-61-8	99.134	197.0442	0.126±0.030	0.068±0.007	0.440±0.06	0.391±0.058	0.088±0.031
n-Propyl gallate	121-79-9	3.043	273.0956	0.089±0.063	0.298±0.015	0.522±0.369	0.224±0.039	0.974±0.168
Ferulic acid ethyl ester	22329-76-6	35.4145	221.0808	0.066±0.005	0.033±0.027	0.469±0.030	0.824±0.337	0.068±0.044
Quercetin	117-39-5	1.787	303.0486	0.047±0.011	0.199±0.019	0.529±0.054	0.001±0.001	0.563±0.104
Quercetin 3'-methyl ether	5041-82-7	154.721	477.0997	0.089±0.009	0.192±0.004	0.471±0.054	0.694±0.141	0.343±0.118
Resveratrol	501-36-0	7.198	289.1129	0.039±0.006	0.268±0.059	0.222±0.112	0.552±0.156	0.905±0.049
Quercetin 3-glucoside	21637-25-2	247.989	463.0844	0.053±0.005	0.251±0.081	0.265±0.087	0.072±0.020	0.424±0.107

Data are mean values of three independent experiments ± standard deviation.

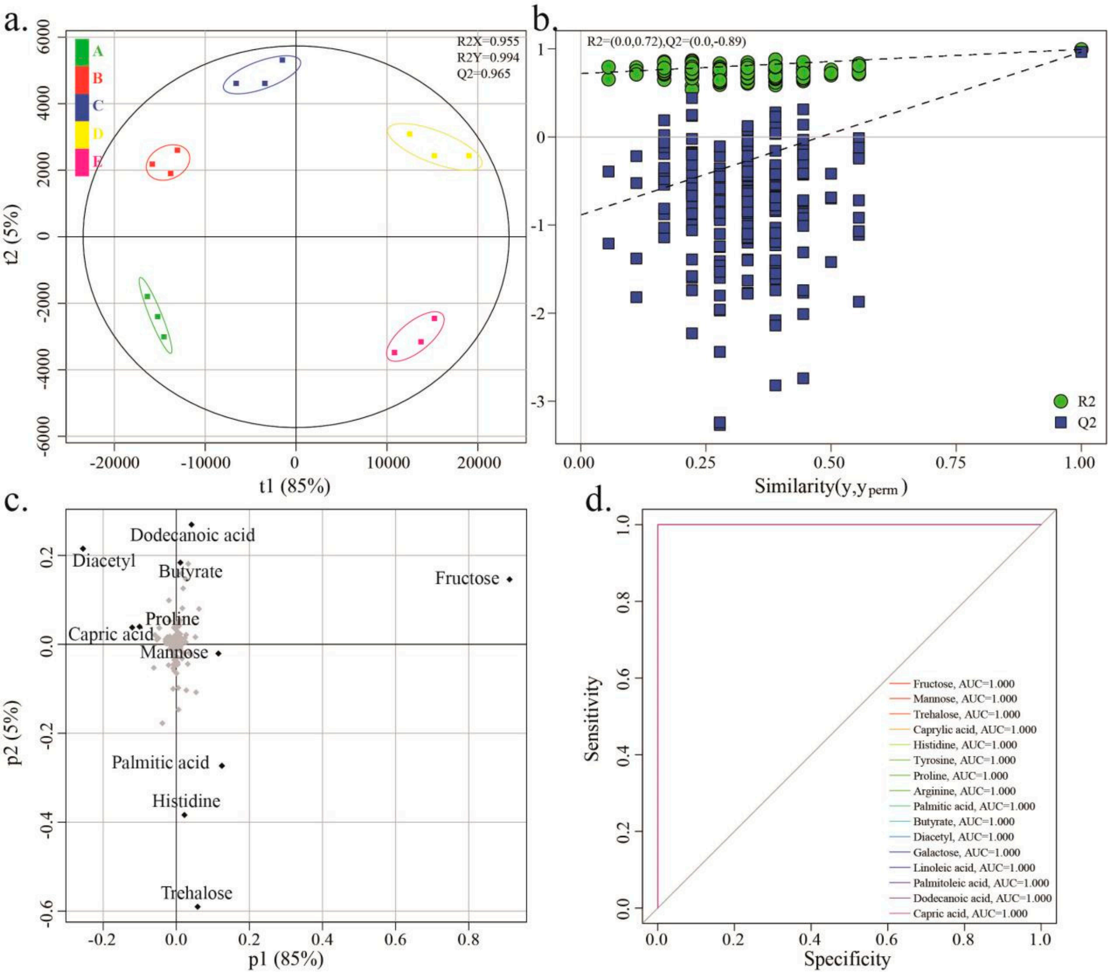

Figure S1. Analysis of differential metabolites during fermentation

Note: In order to estimate the PLS-DA model's stability and prediction capacity, the intercept values of R^2 and Q^2 were obtained after 200 permutations to verify whether the PLS-DA model is beneficial. The differential metabolites were identify by the PLS-DA model combined with VIP projection values (VIP$\geq$1.0) and Student's t-test ($p < 0.05$). The true positive rate is expressed as the ordinate by sensitivity, and the false positive rate is expressed as the abscissa by 1-specificity. The area under ROC curve (AUC) was performed to determine the accuracy of differential metabolites in distinguishing samples. AUC$<$0.5 indicates no discrimination ability; 0.7$\leq$AUC$<$0.9 indicates relatively low distinguishing ability; 0.7$\leq$AUC$<$0.9 indicates relatively high distinguishing ability; AUC$\geq$0.9 indicates high distinguishing ability. a, scatter plot of PLS-DA model; b, 200 permutation tests of PLS-DA model; c, metabolite contribution loading diagram of PLS-DA model; d, ROC analysis of differential metabolites.

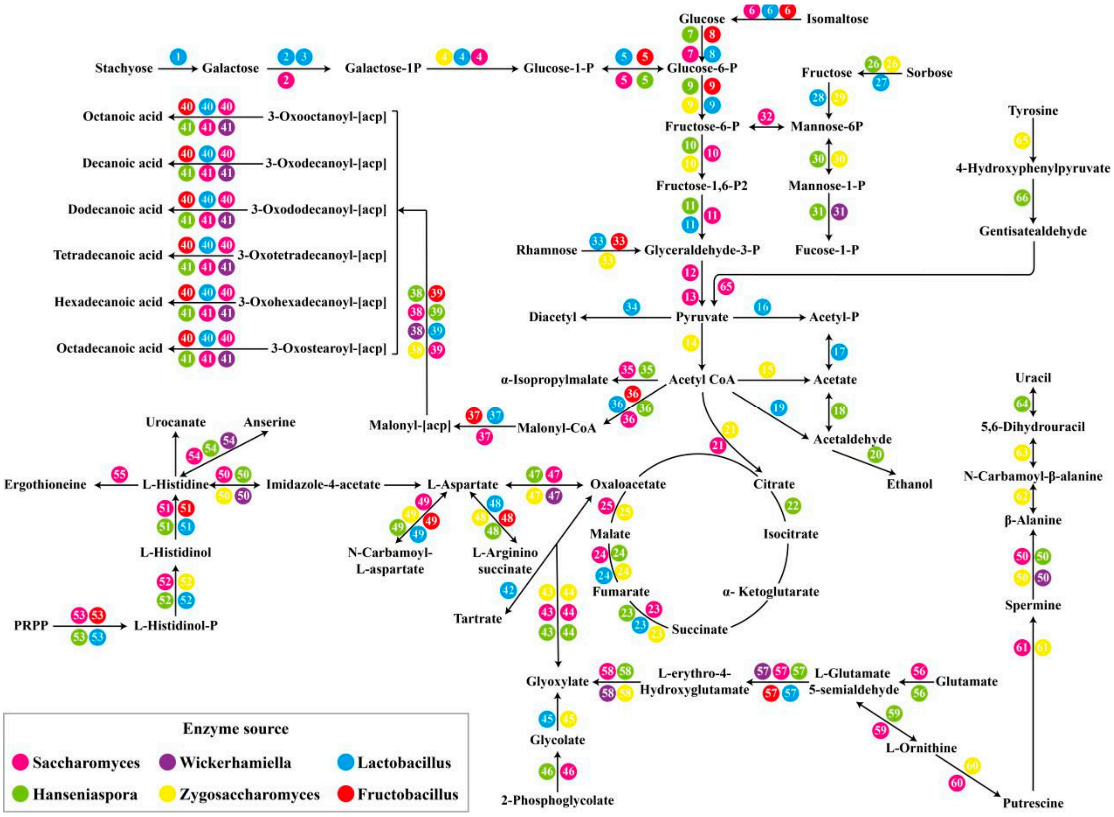

Figure S2. Prediction network diagram of metabolic pathways involved in flavor formation during spontaneous fermentation of Cabernet Sauvignon wine. Note: The primary enzymes involved in flavor formation are indicated in pink, green, purple, yellow, blue and red, which were primarily encoded by Saccharomyces, Hanseniaspora, Zygosaccharomyces, Wickerhamiella, Lactobacillus, and Fructobacillus, respectively. (1, EC 3.2.1.22, alpha-galactosidase; 2, EC 5.1.3.3, galactose mutarotase; 3, EC 2.7.1.6, galactokinase; 4, EC 2.7.7.12, uridyl transferase; 5, EC 5.4.2.2, glucose phosphomutase; 6, EC 3.2.1.10, isomaltase; 7, EC 2.7.1.1, hexokinase; 8, EC 2.7.1.2, glucokinase; 9, EC 5.3.1.9, phosphohexose isomerase; 10, EC 2.7.1.11, phosphohexokinase; 11, EC 4.1.2.13, aldolase; 12, EC 1.2.1.12, triosephosphate dehydrogenase; 13, EC 4.2.1.11, enolase; 14, EC 1.2.7.1, pyruvate synthase; 15, EC 6.2.1.1, acetyl-CoA synthetase; 16, EC 1.2.3.3, pyruvate oxidase; 17, EC 2.7.2.1, acetokinase; 18, EC 1.2.1.3, aldehyde dehydrogenase; 19, EC 1.2.1.10, acetaldehyde dehydrogenase; 20, EC 1.1.1.2, alcohol dehydrogenase; 21, EC 2.3.3.1, citrate (Si)-synthase; 22, EC 4.2.1.3, aconitate hydratase; 23, EC 1.3.5.4, fumarate reductase; 24, EC 4.2.1.2, fumarase; 25, EC 1.1.1.37, malic dehydrogenase; 26, EC 1.1.1.289, sorbose reductase; 27, EC 1.1.1.14, sorbitol dehydrogenase; 28, EC 2.7.1.191, mannose PTS permease; 29, EC 2.7.1.1, hexokinase; 30, EC 5.4.2.8, phosphomannomutase; 31, EC 2.7.7.13, GDP-mannose pyrophosphorylase; 32, EC 5.3.1.8, phosphomannose isomerase; 33, EC 5.3.1.1, phosphotriose isomerase; 34, EC 2.2.1.6, acetolactate synthase; 35, EC 2.3.3.13, alpha-isopropylmalate synthase; 36, EC 6.4.1.2, acetyl-CoA carboxylase; 37, EC 2.3.1.39, [acyl-carrier-protein] S-malonyltransferase; 38, EC 2.3.1.86, yeast fatty

acid synthase; 39, EC 2.3.1.179, beta-ketoacyl-[acyl-carrier-protein] synthase II; 40, EC 1.1.1.100, 3-oxoacyl-[acyl-carrier-protein] reductase; 41, EC 1.3.1.38, trans-2-enoyl-CoA reductase; 42, EC 4.2.1.32, tartrate dehydratase; 43, EC 1.1.1.37, malate dehydrogenase; 44, EC 2.3.3.9, malate synthase; 45, EC 1.1.3.15, (S)-2-hydroxy-acid oxidase; 46, EC 3.1.3.18, phosphoglycolate phosphatase; 47, EC 2.6.1.1, aspartate transaminase; 48, EC 6.3.4.5, argininosuccinate synthase; 49, EC 2.1.3.2, aspartate carbamoyltransferase; 50, EC 1.2.1.3, aldehyde dehydrogenase; 51, EC 1.1.1.23, histidinol dehydrogenase; 52, EC 3.1.3.15, histidinolphosphatase; 53, EC 2.6.1.9, histidinol phosphate aminotransferase; 54, EC 2.1.1.22, carnosine N-methyltransferase; 55, EC 2.1.1.44, L-histidine Nalpha-methyltransferase; 56, EC 1.2.1.88, L-glutamate gamma-semialdehyde dehydrogenase; 57, EC 1.5.1.2, proline oxidase; 58, EC 2.6.1.1, aspartate transaminase; 59, EC 2.6.1.13, ornithine aminotransferase; 60, EC 4.1.1.17, ornithine decarboxylase; 61, EC 2.5.1.16, spermidine synthase; 62, EC 3.5.1.6, beta-ureidopropionase; 63, EC 3.5.2.2, dihydropyrimidinase; 64, EC 1.3.1.1, dihydropyrimidine dehydrogenase; 65, EC 2.6.1.57, aromatic-amino-acid transaminase; 66, EC 1.13.11.27, 4-hydroxyphenylpyruvate dioxygenase; 67, EC 3.7.1.5, acylpyruvate hydrolase).
